# Emergency Awake Laparotomy Using Neuraxial Anaesthesia: A Case Series and Literature Review

**DOI:** 10.3390/jpm14080845

**Published:** 2024-08-09

**Authors:** Matteo Luigi Giuseppe Leoni, Tommaso Rossi, Marco Mercieri, Giorgia Cerati, David Michael Abbott, Giustino Varrassi, Gaetano Cattaneo, Patrizio Capelli, Manuela Mazzoni, Ruggero Massimo Corso

**Affiliations:** 1 Department of Medical and Surgical Sciences and Translational Medicine, Sapienza University of Rome, 29121 Rome, Italy; 2 Department of Anesthesiology and Intensive Care, Guglielmo da Saliceto Hospital, 29122 Piacenza, Italyg.cerati@ausl.pc.it (G.C.); r.corso@ausl.pc.it (R.M.C.); 3 Department of Surgical, Pediatric and Diagnostic Sciences, University of Pavia, 27100 Pavia, Italy; 4 Paolo Procacci Foundation, 00193 Rome, Italy; 5 Emergency Surgery Unit, Guglielmo da Saliceto Hospital, 29122 Piacenza, Italy; 6 General Surgery Unit, Guglielmo da Saliceto Hospital, 29122 Piacenza, Italy; p.capelli@ausl.pc.it

**Keywords:** awake laparotomy, emergency laparotomy, emergency awake laparotomy, neuraxial anaesthesia, lumbar spinal anaesthesia

## Abstract

Emergency laparotomy is a surgical procedure associated with significantly higher mortality rates compared to elective surgeries. Awake laparotomy under neuraxial anaesthesia has recently emerged as a promising approach in abdominal surgery to improve patient outcomes. This study aims to evaluate the feasibility and potential benefits of using neuraxial anaesthesia as the primary anaesthetic technique in emergency laparotomies. We conducted a case series involving 16 patients who underwent emergency laparotomy for bowel ischemia, perforation, or occlusion. Neuraxial anaesthesia was employed as the main anaesthetic technique. We analysed patient demographics, clinical characteristics, intraoperative details, and postoperative outcomes. The primary outcome measures included the adequacy of postoperative pain control, the incidence of postoperative complications, and mortality rates. Among the 16 patients, adequate postoperative pain control was achieved, with only 2 patients requiring additional analgesia. Postoperative complications, including sepsis, wound dehiscence, and pneumonia, were observed in seven patients (44%). The observed mortality rate was relatively low at 6% (one patient). Notably, conversion to general anaesthesia was not necessary in any of the cases, and no early readmissions were reported. Our findings highlight the feasibility and potential benefits of using neuraxial anaesthesia in emergency laparotomies. The observed low mortality rate and the avoidance of conversion to general anaesthesia suggest that neuraxial anaesthesia may be a useful alternative in emergency settings. However, the occurrence of postoperative complications in 44% of patients indicates the need for cautious patient selection and close monitoring. Further research with larger sample sizes is warranted to fully elucidate the efficacy, safety, and potential impact of this technique on patient outcomes in emergency laparotomies.

## 1. Introduction

Emergency laparotomy is a surgical procedure to manage life-threatening conditions affecting the abdominal cavity. This surgery is frequently associated with a mortality rate that is ten times higher than elective surgeries [1]. Different factors can affect the outcome of emergency laparotomy such as age, ASA status, the duration of symptoms, comorbidities, the presence of sepsis and organ dysfunctions [2]. The implementation of early recovery after surgery (ERAS) protocols has led to a significant reduction in morbidity and hospital length of stay for patients undergoing elective surgery [3]. Unfortunately, these pathways are still evolving in emergency surgery and more evidence is needed to improve the outcomes in this heterogeneous group of patients [4]. In emergency surgery, the choice of anaesthesia is crucial for reducing mortality rates. For example, the use of ketamine and dexmedetomidine during general anaesthesia can help improve the outcome of elderly patients in emergency surgery [5].

Very little has been published on the potential use of regional anaesthesia. Awake laparotomies using neuraxial anaesthesia could be an innovative alternative to general anaesthesia for emergency abdominal surgery. In fact, even if abdominal surgeries have been traditionally performed under general anaesthesia, neuraxial anaesthesia has been reported as a possible feasible solution to manage high-risk patients during emergency surgery [6].

In fact, neuraxial anaesthesia has many advantages over general anaesthesia. Firstly, neuraxial anaesthesia results in better pain control. This is particularly important in emergency laparotomies, where effective pain management can significantly impact patient recovery and overall outcomes [7]. Secondly, neuraxial anaesthesia is associated with a lower risk of respiratory complications as it avoids the need for endotracheal intubation and mechanical ventilation [8]. This is particularly beneficial for patients with compromised respiratory function or those at high risk for postoperative pulmonary complications. Furthermore, neuraxial anaesthesia can lead to a more stable hemodynamic profile during surgery [9]. This can be especially advantageous in patients with cardiovascular comorbidities. Additionally, the avoidance of general anaesthesia reduces the presence of cognitive dysfunction and delirium, particularly in the elderly population. Moreover, neuraxial anaesthesia allows for a faster recovery time and can facilitate earlier mobilization and potentially reduce the length of hospital stay. Lastly, the use of neuraxial anaesthesia in emergency laparotomies eliminates the need for airway management. This technique was employed during the COVID-19 pandemic to help prevent aerosolizing the virus during the induction of general anaesthesia and the results demonstrated reduced complications and enhanced recovery [10,11]. In fact, the COVID-19 pandemic had a significant impact on anaesthesia practise in various fields [12]. Though these studies are interesting, further studies are needed to fully understand the safety and potential benefits of this method as an alternative to general anaesthesia in emergency surgeries. In this report, we describe our experience using lumbar neuraxial anaesthesia on a group of patients that underwent emergency laparotomy for bowel ischaemia, perforation with acute peritonitis, or occlusion.

## 2. Materials and Methods

We present a case series of 16 patients who underwent emergency laparotomy for bowel ischaemia, perforation or occlusion between March 2023 and February 2024. Data were collected prospectively, and the report conformed to the ethical standards of the Declaration of Helsinki. All patients provided written consent to authorize the use and disclosure of their health information for the publication of these anonymized data. No ethical approval was required for this case series as our institution’s guidelines do not mandate ethical approval for reporting individual cases or case series.

The clinical characteristics of the patients included in this case series were as follows: all patients were undergoing emergency laparotomy, were aged 18 years or older, and had any range of Body Mass Index (BMI) and American Society of Anesthesiologists (ASA) scores. Neuraxial anaesthesia was not performed in cases where it was contraindicated, including patients with severe spinal deformity or disease, local infection at the puncture site, metastatic spinal disease, coagulopathy, the concurrent use of anticoagulants, or those who refused to undergo neuraxial anaesthesia.

### 2.1. Data Collection

Electronic medical records were used to collect demographic and clinical data. Pre- and intra-operative variables such as age, gender, BMI, comorbidities, surgical diagnosis, previous abdominal surgery, length of hospital stay, intra- and postoperative complications and the duration of surgery were collected. Pain intensity was evaluated postoperatively at 12, 24, 48 and 72 h after surgery with the numeric rating scale (NRS). In patients with peritonitis, the Mannheim Peritonitis Index was calculated to evaluate the mortality risk [13]. This score is calculated for 8 different variables such as the presence of organ failure, diffuse peritonitis, age > 50 years, gender, malignancy, a preoperative duration of peritonitis and the type of abdominal exudate. The maximum total score is 47 and a value > 26 is frequently used as a cut-off value to identify patients at an increased risk of mortality.

### 2.2. Neuraxial Anaesthesia: Technical Procedures

Patients were in the sitting or lateral position depending on patients’ condition to perform neuraxial anaesthesia. Monitoring during and after the surgery and the recovery phase were conducted according to international recommendations [14]. In patients with significant abdominal pain due to peritonitis, a light sedation with ketamine 0.15–0.3 mg/kg/h and midazolam 2 mg was performed as procedural sedation during spinal anaesthesia. Spinal blocks were performed at the L2–L3 level. In 4 patients, an epidural catheter was inserted at the T9–T10 level for postoperative analgesia. This approach was selected for several reasons. Firstly, due to the anticipated long duration of the surgery, continuous pain management was necessary to ensure patient comfort. Secondly, some of these patients were considered at higher risk due to underlying health conditions or advanced age or limitations on the use of rescue medication due to multiple allergies. Additionally, the use of an epidural catheter aimed to mitigate the hemodynamic impact typically associated with spinal anaesthesia.

For all neuraxial anaesthesia, a bolus of 0.5% hyperbaric bupivacaine 8–10 mg+ dexmedetomidine 10 μg+ morphine 100 μg was injected into the subarachnoid space 5–10 min before surgical incision. After spinal injection, patients were placed in a 10–15° Trendelenburg position for a few minutes to increase the spread of the injected spinal solution. When the block reached the T4 level (confirmed by pinprick), the Trendelenburg position was removed, and the patient was considered ready for surgery. Prior to surgical incision, an additional pinprick evaluation at the T4 level was used to assess the adequate level of neuraxial anaesthesia in all patients. All the patients received ondansetron 0.1 mg/kg IV to reduce postoperative nausea and vomiting (PONV) and reduce the incidence of hypotension after spinal anaesthesia [15]. In all patients, sedation during surgery was achieved with small bolus injections of intravenous midazolam, up to 5 mg, and ketamine 0.15–0.3 mg/kg/h, targeting a RASS score of 0 to −2. Intraoperative fluid infusion was based on goal-directed fluid therapy [16,17].

### 2.3. Statistical Analysis

Due to this study design, only a descriptive statistical analysis was conducted. Continuous variables are reported as the median and interquartile range while categorical data as relative number and percentage. The Shapiro–Wilk test was used to evaluate data distribution. The Friedman test with Dunn’s correction for multiple comparisons was used to evaluate non-normal distributed repeated measurements. R software v4.2.2 (R Foundation for Statistical Computing, Vienna, Austria, www.r-project.org) was used for the analyses.

## 3. Results

Sixteen patients were included in this study. Demographic and clinical variables are reported in Table 1. Ten patients were female (62.5%), while six patients (37.5%) were male. The median age was 75 years old (65–84.5 IQR) and the median BMI was 22.8 kg/m^2^ (20.8–26.6 IQR). Five patients (31.2%) were classified as ASA 2, eight patients (50%) as ASA 3, and the remaining three patients (18.8%) as ASA 4. A significant number of patients had comorbidities, including hypertension (eight patients), COPD (six patients), and diabetes type II (four patients). Eight patients (50%) underwent previous abdominal surgery before the emergency laparotomy, while the other eight patients (50%) had no previous abdominal surgery. The diagnoses leading to emergency laparotomy included bowel perforations, volvulus, and bowel obstructions due to adhesions or hernias. Specific surgical interventions ranged from segmental resections, such as sigmoid colectomy and ileal resection, to more complex procedures like subtotal colectomy and Hartmann procedures. The median length of hospital stay was 11 days (8–25 IQR) and the median duration of surgery was 119 min (80–140 IQR). Notably, neuraxial anaesthesia was used as the primary anaesthetic technique in all cases, while in four patients (25%), an epidural catheter was placed to provide continuous pain management.

**Figure 1:**
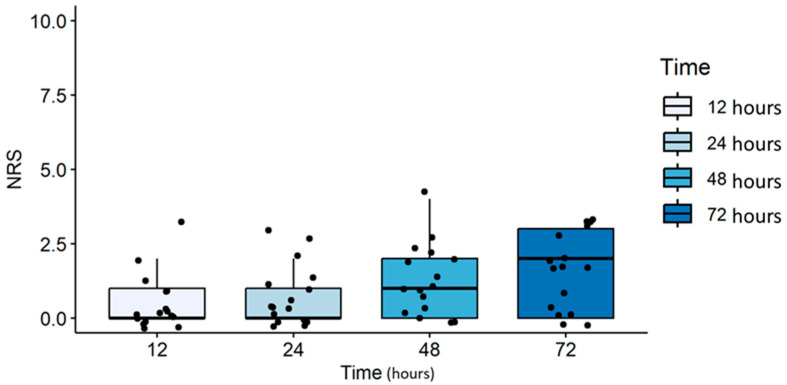
Postoperative pain intensity (NRS) at 12, 24, 48 and 72 h after surgery. Effective pain control was achieved postoperatively from 12 to 72 h after surgery. Although there was a gradual and statistically significant increase in NRS over this period (*p* = 0.014), the median NRS values remained below the threshold that would require additional analgesic intervention.

An adequate control of pain was obtained during the postoperative period, from 12 to 72 h after surgery. A gradual increase in NRS values was found from 12 to 72 h after surgery, and though the observed trend was statistically significant (*p* = 0.014), median NRS values were below the cut-off for the use of analgesic rescue medications (Figure 1). Only two patients (12%) required the postoperative intravenous administration of rescue medications (paracetamol or ketorolac) for postoperative analgesia. No intraoperative hypotension and no complications (nausea, vomiting, coughing or discomfort) were observed. Only one patient (6%) required atropine injection for a transient bradycardia. Surgical relaxation was reported as adequate by most (92%) of the operating surgeons.

Seven patients (44%) had postoperative complications. Three patients (19%) developed sepsis and septic shock and two patients (12%) developed a surgical wound dehiscence requiring reoperation. One patient (6%) developed postoperative pneumonia. Three patients (19%) required postoperative ICU admission. One of them required admission to the ICU postoperatively for a few hours, without the need of mechanical ventilation or vasopressors. One patient was admitted to the ICU with a temporary open abdomen due to bowel ischemia, necessitating a second surgery. Subsequently, this patient was intubated in the ICU without complications, and the second surgery was performed 48 h later. The third patient was admitted to the ICU with septic shock caused by anastomotic dehiscence five days after surgery, necessitating mechanical ventilation and the infusion of vasopressors. Unfortunately, this patient died in the ICU 9 days after surgery. In summary, fifteen patients (94%) were discharged without postoperative symptoms. None of the patients reported a post-dural puncture headache and no neurological sequelae were observed throughout the postoperative period until discharge. The observed median Mannheim peritonitis index was 25 (21–37 IQR) in nine patients (56%) with peritonitis and the median estimated mortality was 26% (16–64 IQR). Regardless of the predicted mortality, the observed mortality rate was 6%.

## 4. Discussion

This study suggests the feasibility of lumbar spinal anaesthesia as the main anaesthetic technique for emergency laparotomy in patients with bowel ischaemia, perforation, or occlusion. In fact, our data show that spinal anaesthesia was technically feasible and associated with good intra- and postoperative outcomes. Adequate pain control was achieved during the postoperative period, with minimal need for rescue analgesia. Furthermore, the absence of intraoperative complications and the low incidence of adverse events highlight the safety profile of neuraxial anaesthesia in emergency laparotomy. In high-risk and older patients, the need for rescue analgesia in the postoperative period can pose several significant challenges. These patients often have multiple comorbidities that increase the risks of complications and adverse effects such as hemodynamic instability, gastrointestinal disturbances, renal impairment and postoperative cognitive dysfunction or delirium [18]. Additionally, older patients frequently exhibit altered pharmacokinetics and pharmacodynamics, making it difficult to predict the efficacy and safety of standard dosages of analgesic medications [19]. These complications can prolong hospital stays and recovery times, adding to the overall burden on both the patient and the healthcare system [20].

### 4.1. Advantages of Spinal Anaesthesia

Spinal anaesthesia has many potential advantages over general anaesthesia. These advantages include a rapid onset, a better suppression of stress response with reduced negative cardiocirculatory effects, a deep sensory and motor block, the avoidance of tracheal intubation and a decreased need for postoperative analgesics [7]. All these advantages are particularly useful in elderly and critically ill patients undergoing emergency surgery. Moreover, neuraxial anaesthesia is associated with a faster recovery of gastrointestinal transit after surgery, a reduced incidence of PONV, decreased intraoperative blood loss and the earlier mobilization of patients, with a resultant global cost reduction [3,21]. This approach not only enhances patient comfort and recovery but can also optimizes the utilization of medical resources in countries with limited healthcare resources or in areas with restricted access to medical facilities and supplies.

Locoregional anaesthesia is also associated with reduced postoperative pulmonary and neurocognitive complications and reduced postoperative intensive care admission [22]. By avoiding the use of volatile anaesthetics and minimizing systemic opioid exposure, patients may experience shorter hospital stays and improved overall outcomes [23]. Consequently, neuraxial anaesthesia has been suggested as an alternative to general anaesthesia in high-risk surgical patients undergoing elective abdominal surgery [8]. Indeed, recent articles show the use of spinal anaesthesia or continuous spinal anaesthesia at the thoracic level for various surgeries including laparoscopy [24,25,26,27,28]. However, spinal anaesthesia or continuous spinal anaesthesia was very rarely used for emergency surgery. A significant increase in the use of neuraxial anaesthesia for abdominal surgery was observed only during the COVID-19 pandemic to reduce droplet spread during airway manipulation. For this reason, the Royal College of Anaesthetists promoted the use of regional anaesthesia during the pandemic [29].

Moreover, regional anaesthesia has been recently suggested for postoperative pain management in transplant surgery [30]. Unfortunately, the adoption of this practise often depends on the specific transplant centre or the discretion of individual practitioners. The use of combined spinal–epidural anaesthesia was recently suggested in a case report of robotic liver resection for hepatocellular carcinoma in a patient with severe comorbidities [31].

### 4.2. Considerations for Emergency Laparotomy

Emergency laparotomy is a surgical procedure performed for specific life-threatening situations such as bowel perforation, intestinal obstruction, traumatic injuries, or acute abdominal pain of uncertain origin. Unlike elective procedures, emergency laparotomy requires immediate intervention to prevent further complications. Different critical elements should be considered while treating patients undergoing emergency laparotomy and the comprehensive management of pre-, intra-, and postoperative care is essential [32].

The presence of peritonitis can potentially induce caution and necessitate additional considerations when deciding to perform neuraxial anaesthesia. In fact, the peritonitis often results in significant physiological changes and can complicate the anaesthetic management. Firstly, the inflammatory response associated with peritonitis can lead to systemic effects such as fever, tachycardia, and hypotension. Moreover, the risk of infection spreading via a spinal needle in an already infected peritoneal cavity is a concern that must be addressed. However, recent reports indicate that the incidence of central nervous system infection following neuraxial puncture in patients with ongoing bacteraemia is very low, ranging from 0.007% to 0.6% [33,34]. Ensuring strict aseptic techniques is mandatory. Lastly, the patient’s overall condition, including hemodynamic stability, coagulation status, and the absence of septic shock, must be thoroughly assessed before opting for neuraxial anaesthesia. While neuraxial anaesthesia can be considered for patients with peritonitis, it requires a careful evaluation of the patient’s condition and meticulous planning to mitigate risks and ensure the best possible outcomes.

Considering the balance of benefits and risks, we believe that neuraxial anaesthesia can be a viable alternative for patients with perforation peritonitis who have a high risk of complications from general anaesthesia. This is particularly applicable when there is no septic shock, hemodynamic parameters are stable, and coagulation is normal.

### 4.3. Neuraxial Anaesthesia for Abdominal Surgery

In 2013, the first case report of the use of spinal anaesthesia for urgent laparotomy in a patient with severe myasthenia gravis was published [35]. The patient required urgent laparotomy for ileal perforation due to a 2.5 cm foreign body in the terminal ileum. After spinal anaesthesia at the L2–L3 level with 8 mg of 0.5% hyperbaric bupivacaine and 20 μg of fentanyl, the patient underwent a 115 cm ileectomy with mechanical ileocecal anastomosis. No adverse respiratory events or hemodynamic instability was observed, and the patient was successfully discharged 12 days after surgery. Romanzi et al. [6] recently reported the feasible use of neuraxial anaesthesia in patients undergoing awake laparotomy. The authors included 43 patients requiring urgent abdominal surgery and 27 cases of elective abdominal surgery. Neuraxial anaesthesia was performed via combined spinal epidural or spinal anaesthesia in 35.7% and 30% of patients, respectively, while 34.3% underwent epidural anaesthesia. Hyperbaric bupivacaine (10 mg at 0.5%) and morphine sulphate (100–150 mcg) were injected in the subarachnoid space. Sedation was necessary in 24.3% of patients during surgery and 5.7% required a conversion to general anaesthesia. Unfortunately, the authors did not report the level of spinal injection nor the level to which the anaesthetic arrived after allowing it to spread in the cranial direction. Consequently, no direct comparisons with our data are possible. In 2020, the same authors published another article during the first wave of the COVID-19 pandemic, including thirteen high-risk (ASA score  ≥ 3) patients who required emergency laparotomy [10]. Surgery was performed under different anaesthetic management including combined spinal epidural anaesthesia, spinal anaesthesia or epidural anaesthesia. Spinal anaesthesia was mainly performed at L2–L3 or L3–L4 levels with hyperbaric 0.5% bupivacaine and morphine sulphate. Most of the included patients underwent combined spinal epidural anaesthesia along with additional sedation. The authors reported good intra- and postoperative outcomes, supporting the possible use of regional anaesthesia for awake laparotomy. Farda et al. [36] reported the use of spinal anaesthesia for emergency laparotomy in Kabul, Afghanistan, in a place characterized by challenging conditions and healthcare limited resources. This article represents the most significant publication on the topic, since 196 patients underwent emergency laparotomy with spinal anaesthesia at the L2–L3 or L3–L4 level. The authors used 15 mg of bupivacaine, injected in the subarachnoid space without other adjuvants such as morphine or fentanyl. However, a high incidence of hypotension (12.7%) was reported. Combined spinal epidural anaesthesia was used by Pereira et al. [37] and Niraj et al. [38] for emergency laparotomy. The authors injected small amounts of 0.5% hyperbaric bupivacaine followed by small doses of 0.5% isobaric levobupivacaine until a T6 dermatomal block was reached. Even if this technique was considered feasible and effective, a high risk of conversion to general anaesthesia due to accidental displacement of the spinal catheter was reported.

One of the most prevalent indications for considering regional anaesthesia is the respiratory function of the patient. Patients with significant underlying respiratory disease have a greater risk of prolonged postoperative ventilation following general anaesthesia [35]. Almost 70% of our patients were classified as ASA 3 or 4 due to multiple comorbidities and COPD was the most common comorbidity.

A recent publication showed that thoracic epidural anaesthesia could be another feasible option for patients with severe pulmonary disease requiring an awake emergency laparotomy for bowel ischaemia in the absence of postoperative intensive care monitoring [39]. Though this is certainly an interesting alternative to general anaesthesia and neuraxial anaesthesia, the article is only a single-patient case study.

### 4.4. Our Proposed Approach

We used neuraxial anaesthesia in patients with acute abdominal pathology requiring urgent surgical intervention (bowel ischaemia, perforation with acute peritonitis, or occlusion). No patient required conversion to general anaesthesia. We decided to perform spinal anaesthesia with low-dose hyperbaric bupivacaine (8–10 mg) because it has a more predictable cephalad spread for patients in the Trendelenburg position after spinal injection [40]. Moreover, using the Trendelenburg position immediately after injection is advantageous because it ensures venous return, thereby maintaining cardiac output and blood pressure. We decided to add dexmedetomidine as an adjuvant since it can shorten the onset time of spinal anaesthesia, prolong the block duration, and decrease the occurrence of shivering [41,42]. None of the patients experienced nausea and vomiting during surgery, probably due to the administration of pre-emptive antiemetics and the lack of postoperative opioids. In four patients, a thoracic epidural catheter was combined with subarachnoid anaesthesia. In the first patient of our case series, the epidural catheter was added as a rescue therapy to potentially manage intra- and postoperative pain due to a lack of sufficient experience in managing emergency laparotomy with spinal anaesthesia alone. In two patients, the epidural catheter was inserted due to the presence of significant comorbidities requiring lower doses of local anaesthetic in the subarachnoid space or due to the expected surgical complexity with potentially intense postoperative pain. In the fourth patient, an epidural catheter was inserted to enhance postoperative pain management, given the limitations on the use of rescue medication due to multiple allergies, including non-steroidal anti-inflammatory drugs.

During emergency laparotomy, several intraoperative complications may arise that necessitate prompt and effective management. One potential complication is the prolonged duration of surgery, which may lead to the end effect of neuraxial anaesthesia. In such cases, if an epidural catheter is in place, a top-up injection of local anaesthetics can be administered to extend the duration of analgesia. If an epidural catheter is not in place, alternative strategies must be employed. This can include administering supplemental intravenous analgesics or converting to general anaesthesia if the duration of surgery needs to be significantly extended or if a patient is unable to protect their airway. Massive bleeding, although rare during emergency laparotomy for bowel surgery, can pose a significant challenge. If this occurs, it is crucial to manage hemodynamic stability through rapid fluid resuscitation, blood transfusion, and close monitoring. These strategies highlight the importance of preparedness and adaptability in managing neuraxial anaesthesia during emergency laparotomy.

Our preliminary data suggest a possible role of spinal anaesthesia as the main anaesthetic technique for emergency laparotomy, though this topic necessitates careful considerations. Such considerations include patient selection criteria, anatomical difficulties, procedural feasibility, standardized protocols, and anaesthesia provider expertise. Consequently, the safety and efficacy of spinal anaesthesia in emergency awake laparotomy require further investigation through well-designed prospective studies with standardized protocols and comparative analyses against traditional techniques. Moreover, further studies with long-term follow-up are needed to better evaluate the feasibility of spinal anaesthesia in emergency laparotomy and to confirm these preliminary findings.

If we apply the SANRA (Scale for the Assessment of Narrative Review Articles) [43] to evaluate the quality of our narrative review, our article achieves a score of 9 (the justification of the article’s importance for the readership = 2, a statement of concrete aims or the formulation of questions = 2, a description of the literature search = 0, referencing = 2, scientific reasoning = 1, and an appropriate presentation of data = 2). While the SANRA scale does not have established cut-offs for grading review quality beyond the threshold of 4 or below, which indicates poor quality, a score of 9 suggests that our review meets high standards.

### 4.5. Limitations

Despite the insights provided by this study, several limitations warrant consideration. First, the small sample size and single-centre nature of the study may limit the generalizability of these findings. Second, the absence of a control group receiving general anaesthesia precludes direct comparisons of outcomes between different anaesthesia modalities. Moreover, the lack of long-term follow-up limits the comprehensive assessment of long-term outcomes following emergency laparotomy under spinal anaesthesia. Furthermore, while lumbar spinal anaesthesia shows promise as an alternative anaesthetic technique, its applicability may be constrained by patient-specific factors, anatomical considerations, and the expertise of anaesthesia providers as previously reported [44]. In addition, the exclusion of patients with severe spinal deformity or disease and specific contraindications to spinal anaesthesia may introduce selection bias and limit the external validity of this study. Finally, another significant limitation of this study is the lack of a comprehensive anaesthetic risk assessment using validated scoring systems such as the Portsmouth Physiologic and Operative Severity Score for the Enumeration of Mortality and Morbidity (P-POSSUM) [45], the American College of Surgeons National Surgical Quality Improvement Program (ACS-NSQIP) [46], or the Charleston Comorbidity Index 2 [47]. Unfortunately, the quantification of these scores was not feasible due to missing variables.

## 5. Conclusions

Our preliminary data support the use of lumbar spinal anaesthesia as a viable alternative to general anaesthesia for emergency laparotomy in patients with bowel ischemia, perforation with acute peritonitis, or obstruction. No intraoperative complications were observed, and an adequate control of pain was achieved during the postoperative period. None of the patients reported a post-dural puncture headache and no neurological sequelae were observed during the postoperative period. Future prospective studies with an adequate sample size are needed to confirm these preliminary findings and to elucidate optimal patient selection criteria and procedural protocols for maximizing the benefits of awake laparotomy under spinal anaesthesia.

## Figures and Tables

**Table 1:** The table presents demographic and clinical data for 16 patients who underwent emergency laparotomy under neuraxial anaesthesia (NA).

Variable	Age	Gender	ASA	Comorbidities	Previous Abdominal Surgery	Diagnosis and Surgery Performed	Anaesthesia	Surgery Duration (min)	Postoperative Complications and Final Outcome
Patient 1	41	M	2	None	No	Perforated diverticulitis. Sigmoid colectomy + protective ileostomy	NA + EA	133	No complications. Discharged
Patient 2	33	F	2	None	Yes	Biliary Peritonitis. Ileal resection + ileo-ileal anastomosis.	NA	75	No complications. Discharged
Patient 3	71	M	2	None	No	Bowel perforation. Subtotal colectomy with ileosigmoid anastomosis.	NA	170	Postoperative sepsis. ICU admission. Discharged
Patient 4	73	F	3	Hypertension, hypothyroidism	Yes	Anastomotic leakage. Left colectomy + protective ileostomy.	NA	182	Postoperative sepsis. ICU admission. Death 9 days after surgery
Patient 5	72	M	3	Stroke, COPD	Yes	Anastomotic leakage. Ileocolic resection + Ileocolic anastomosis.	NA	123	Surgical wound dehiscence. Discharged
Patient 6	85	M	3	Hypertension	Yes	Bowel obstruction. Lysis of adhesions.	NA	83	No complications. Discharged
Patient 7	77	M	3	Obesity, diabetes type II, COPD	No	Volvulus. Sigmoid colectomy + protective ileostomy.	NA	81	No complications. Discharged
Patient 8	60	F	2	None	No	Bowel obstruction. Right hemicolectomy	NA	75	No complications. Discharged
Patient 9	85	F	4	COPD, hypertension	No	Perforated diverticulitis. Hartmann procedure.	NA	119	Postoperative sepsis. ICU admission. Discharged
Patient 10	89	F	4	Dilatative cardiomyopathy (EF 20%), fibrotic interstitial disease, hypothyroidism, Parkinson disease	Yes	Volvulus. Ileal resection + ileo-ileal anastomosis.	NA + EA	95	No complications. Discharged
Patient 11	84	F	3	Hypertension	No	Left colon perforation + vaginal fistula. Hartmann procedure.	NA + EA	140	No complications. Discharged
Patient 12	77	M	3	Obesity, diabetes type II, COPD	Yes	Bowel obstruction. Lysis of adhesions + ileostomy.	NA	110	Surgical wound dehiscence. Discharged
Patient 13	84	F	3	COPD, hypertension	No	Right colon perforation. Right colectomy with ileotransverse astomosis + sigmoid colectomy with colostomy.	NA	120	Pneumonia. Discharged
Patient 14	90	F	4	Obesity, diabetes type II, hypertension, COPD	Yes	Bowel obstruction. Lysis of adhesions.	NA	44	No complications. Discharged
Patient 15	48	F	3	tracheomalacia, renal tubulopathy, diabetes type 2, hypertension, asthma, myocardial fibrosis (preserved EF), sarcoidosis, Bechet vasculitis	Yes	Left colon perforation. Left colectomy + protective ileostomy.	NA + EA	160	No complications. Discharged
Patient 16	70	F	2	Hypertension	No	Volvulus. Ileal resection.	NA	80	Bowel ischemia. ICU admission. Discharged.

Abbreviation: F, female; M, male; ASA, American Society of Anaesthesiologists (ASA) score; COPD, chronic obstructive pulmonary disease; NA, neuraxial anaesthesia EA, epidural analgesia. ICU, intensive care unit.

## Data Availability

The data analysed during the current study are available from the corresponding author on reasonable request.
